# Supplementary material for: Use of Cannabis and Cannabinoids for Treatment of Cancer
Source: Cancers (Basel). 2022 Oct 20;14(20):5142. doi: 10.3390/cancers14205142 (PMC9600568; doi:10.3390/cancers14205142)
Supplement: Supplementary file 1 [file cancers-14-05142-s001.zip › cancers-1926564-supplementary.pdf]

Supplementary Table S1. Clinical trials related to cannabinoid use in cancer (source clinicaltrials.gov as of 06.22.2022).

| Official title | Condition or disease | Comparison group/Cohort | Outcome | Identifier |
|---|---|---|---|---|
| Palliative care | | | | |
| Inhaled PPP001 Versus Immediate-release Oral Opioids for the Management of Breakthrough Pain in Cancer Subjects: a Randomized, Open-Label, Crossover, Comparison Study | Cancer<br>Breakthrough Cancer Pain | Experimental Drug: PPP001 (inhaled cannabinoids)<br>Active Comparator: Morphine sulfate or Hydromorphone or Oxycodone | Recruiting | NCT03564548 |
| Safety And Efficacy Of Ppp001-kit For The Uncontrolled Pain Relief In Patients With Advanced Cancer: A Randomized, Double-Blind, Placebo-Controlled, Parallel Group Study | Cancer Pain<br>Quality of Life<br>Pain,<br>Acute Cannabis Use | Drug: PPP001 (cannabis dosing capsule with THC/CBD) inhalation with a device<br>Drug: Placebo | Recruiting | NCT04042545 |
| Patient Reported Symptom Control With THC and CBD Use | Cancer-associated Pain<br>Hematopoietic and Lymphoid Cell Neoplasm<br>Malignant Solid Neoplasm | Electronic Health Record Review<br>Questionnaire Administration | Recruiting | NCT04875286 |
| Quantification of Endo- and Phytocannabinoids With Comparison to Pain Medication Requirements and Surgical Outcomes for Patients Undergoing Abdominal Surgery for Cancer | Post-Surgical Pain<br>Post-surgical complication<br>Cancer<br>Cannabis use | Daily cannabis users<br>Cannabis non-users | Recruiting | NCT04988490 |
| Multicenter, Randomized, Double-blind, Placebo-controlled, Phase III Clinical Trial to Investigate the Efficacy and Safety of Dronabinol in the Improvement of Chemotherapy-induced and Tumor-Related Symptoms in Patients With Locally Advanced or Metastatic Pancreatic Cancer During First-line Chemotherapy (DIsCOvER) | Pancreatic Cancer Non-resectable<br>Chemotherapy-induced Nausea and Vomiting<br>Pancreatic Cancer Metastatic | Drug: Dronabinol in Oral Dosage Form<br>Drug: Placebo | Recruiting | NCT03984214 |

| | | | | |
|---|---|---|---|---|
| Treatment of Established Chemotherapy-Induced Neuropathy With N-Palmitoylethanolamide, a Cannabimimetic Nutraceutical: A Randomized Double-Blind Phase II Pilot Trial | Chemotherapy-Induced Peripheral Neuropathy Hematopoietic and Lymphoid Cell Neoplasm Malignant Solid Neoplasm | Drug: Palmidrol Drug: Placebo Administration Other: Quality-of-Life Assessment | Recruiting | NCT05246670 |
| NanaBis™ an Oro-buccal Administered Equimolar delta-9-THC & CBD Formulation as Monotherapy for Management of Opioid Requiring Bone Pain Due to Metastatic Cancer Phase 3 Multi Centre Blinded Randomized Withdrawal Active & Placebo Controlled Trial | Cancer-related pain | Drug: NanaBis™<br>Drug: Oxycodone CR<br>Drug: Placebo Spray<br>Drug: Placebo Tablet<br>Drug: Oxycodone IR | Not yet recruiting | NCT04808531 |
| Pharmacokinetic and Pharmacodynamic Evaluation of 3 Standard Formulations of Δ9-THC in Healthy Volunteers and Post-chemotherapy Patients in Colombia | Nausea<br>Post-Chemotherapy Vomiting<br>Cancer Pain Cancer- Related Pain Neoplasm | Drug: Pharmacokinetic and pharmacodynamic profile of THC oral formulations with Dronabinol as comparator | Not yet recruiting | NCT05272865 |
| A Randomized, Double-Blind, Placebo-Controlled, Multiple Crossover N-of-1 Study Design of the Use of Medicinal Cannabis Oil-Based Extracts for Symptom Management in Cancer Patients | Pain<br>Nausea<br>Anxiety<br>Sleep Disturbance | Drug: Cannabis | Active, not recruiting | NCT03948074 |
| A Double-blind, Randomized, Placebo Controlled Clinical Trial to Evaluate the Efficacy of Orexigenic Therapy With Delta-9-tetrahydrocannabinol in Advanced Cancer Patients With Chemosensory Abnormalities - a Pilot Study | Cancer<br>Anorexia<br>Taste Disorders<br>Olfactory Disorders | Drug: Marinol (Dronabinol) Marinol is being used as an appetite stimulant for treatment of cancer-induced anorexia | Completed (no results) | NCT00316563 |
| A Pilot Trial to Evaluate Syndros in Decreasing Opioid Requirement in Patients With Bone Metastases From Breast Cancer | Bone Metastases<br>Breast Cancer<br>Pain | Drug: Syndros (Dronabinol) | Completed (no results) | NCT03661892 |
| A Double Blind, Randomized, Placebo Controlled, Parallel Group Dose-range Exploration | Palliative Care<br>Pain<br>Cancer | Drug: Sativex Low Dose<br>Drug: Sativex Medium Dose | No baseline differences | NCT00530764 |

| | | | | |
|---|---|---|---|---|
| Study of Sativex® in Relieving Pain in Patients With Advanced Cancer, Who Experience Inadequate Analgesia During Optimized Chronic Opioid Therapy | | Drug: Sativex High Dose | across the groups. Demonstrated efficacy and safety in low- and medium doses (238) | |
| Randomized, Double-Blind, Placebo-Controlled Trial of Palonosetron/Dexamethasone With or Without Dronabinol for the Prevention of Chemotherapy-Induced Nausea and Vomiting After Moderately Emetogenic Chemotherapy | Chemotherapy-induced Nausea and Vomiting Unspecified Adult Solid Tumor | Arm 1<br>Drug: Dexamethasone<br>Drug: Dronabinol<br>Drug: Palonosetron Hydrochloride<br>Arm 2<br>Drug: Dexamethasone<br>Drug: Palonosetron Hydrochloride<br>Drug: Placebo | Completed (no publications) | NCT00553059 |
| A Multicenter, Non-comparative, Open-label Extension Study to Assess the Long Term Safety of Sativex Oromucosal Spray (Sativex; Nabiximols) as Adjunctive Therapy in Patients With Uncontrolled Persistent Chronic Cancer-Related Pain | Pain<br>Advanced Cancer | Drug: Nabiximols | Completed (no publications) | NCT01337089 |
| A Double Blind, Randomized, Parallel Group, Placebo Controlled, Comparative Study of the Efficacy, Safety and Tolerability of Cannabis Based Medicine (CBM) Extracts in Patients With Cancer-related Pain | Palliative care<br>Pain<br>Cancer | Drug: Placebo<br>Drug: Sativex<br>Drug: THC Alone | The mean pain Numerical Rating Scale score was statistically significant for THC/CBD group in pain reduction (239) | NCT00674609 |
| An Open-label, Extension Study, to Investigate the Long-term Safety and Tolerability of Cannabis Based Medicine Extracts in Patients With Cancer-related Pain | Pain<br>Cancer | Drug: Sativex<br>Drug: GW-2000-02 (THC alone) | In mean Brief Pain Inventory-Short Form Scores for "pain severity" and "worst pain" showed an | NCT00675948 |

| | | | | |
|---|---|---|---|---|
| | | | improvement in the THC/CBD spray patients(240) | |
| The Effect of Medical Cannabis Inpatients With Palliative Pancreatic Cancer | Neoplasms Pancreatic Cachexia Cancer Cannabis Appetite Loss Palliative Medicine Morbidity Mortality | Drug: THC and CBD | Unknown | NCT03245658 |
| Cancer treatment | | | | |
| Effects of Cannabis Use in Cancer Patients: A Feasibility Study | Solid Tumor Adult | Oral Cannabis | Recruiting | NCT03617692 |
| A Pilot Study to Assess the Role of Cannabis Added to the Supportive Care Regimen During Chemoradiation for Head and Neck Cancer | Head and Neck Cancer | Observational group using THC/CBD formulations | Recruiting | NCT03431363 |
| Outcomes Mandate National Integration With Cannabis as Medicine (200,000 participants) | Chronic Pain Fibromyalgia Seizures Hepatitis Cancer Crohn Disease HIV/AIDS Multiple Sclerosis Traumatic Brain Injury Sickle Cell Disease Post-Traumatic Stress Disorder Tourette Syndrome Ulcerative Colitis Glaucoma Epilepsy Inflammatory Bowel Diseases Parkinson Disease Amyotrophic Lateral Sclerosis Chronic Traumatic Encephalopathy Anxiety Depression Insomnia | Drug: Cannabis, Medical Device: RYAH-Medtech Inhaler | Recruiting | NCT03944447 |

| | | | | |
|---|---|---|---|---|
| | Autism<br>Opioid-use Disorder<br>Bipolar Disorder<br>Covid | | | |
| Medical Marijuana in the Pediatric Central Nervous System Tumor Population | Central Nervous System Tumor<br>Brain Tumor<br>Spinal Tumor | Drug: Medical Marijuana | Active, not recruiting | NCT03052738 |
| Phase Ib, Open-label, Multicenter, Intrapatient Dose-escalation Clinical Trial to Assess the Safety Profile of the TN-TC11G (THC+CBD) Combination With Temozolomide and Radiotherapy in Patients With Newly-diagnosed Glioblastoma | Glioblastoma | Drug: TN-TC11G Drug: Temozolomide<br>Radiation: Radiotherapy | Not yet recruiting | NCT03529448 |
| Expression of G Protein-coupled Receptors and Ligands in Inflammatory Bowel Diseases and Colon Cancer | Ulcerative colitis<br>Crohn's Disease<br>Colon Cancer | Ulcerative colitis group<br>Crohn's Disease group<br>Colon Cancer group<br>Control group (healthy individuals) | Completed (no results) | NCT02735941 |
| A Phase I, Sequential Cohort, Open-Label, Dose-escalation Study of the Safety and CNS Pharmacokinetics of Dexanabinol in Patients With Brain Cancer | Brain cancer | Drug: Dexanabinol | Completed (no results) | NCT01654497 |
| A Pilot Study of Dronabinol for Adult Patients With Primary Gliomas | Brain Neoplasms<br>Nausea<br>Vomiting | Drug: Dronabinol | Completed (no publications) | NCT00314808 |
| A Phase 1, Pharmacokinetically-Guided, Dose Escalation Study to Assess the Safety and Tolerability of Dexanabinol in Patients With Advanced Solid Tumors | Solid Tumor | Drug: Dexanabinol<br>Other: Cremophor | Progression-free survival increased | NCT01489826 |
| A Two Part Study to Assess the Tolerability, Safety and Pharmacodynamics of Sativex in Combination With Dose-intense Temozolomide in Patients With Recurrent Glioblastoma | Glioblastoma multiforme | Drug: Temozolomide and Sativex | Increased 1-year survival rate in 39% (185) | NCT01812603; NCT01812616 |
| A study: Pure CBD as single agent for solid tumors | Solid tumor | Drug: CBD | (241) | NCT02255292 |
| A Phase 1b Study to Assess the Safety and Anti-tumour Activity of Dexanabinol Monotherapy and | Hepatocellular carcinoma<br>Pancreatic cancer | Drug: Dexanabinol<br>Drug: Sorafenib<br>Drug: Nab-paclitaxel | Unknown | NCT02423239 |

| | | | | |
|---|---|---|---|---|
| Dexanabinol in Combination With Chemotherapy in Patients With Advanced Tumours | | Drug: Gemcitabine | | |
| A Phase I Study of BRCX014 to Investigate Dose-Ranging Safety and Pharmacokinetics in Adults With Glioblastoma (GBM) and Non-Methylated MGMT Gene Status | Glioblastoma | Drug: Temozolomide<br>Drug: BRCX014 (cannabinoid formulation) | Unknown | NCT03687034 |
| Randomized Double-Blind, Placebo-Controlled Parallel Multi-Center Study to Assess the Efficacy of Cannabidiol (BRCX014) Combined With Standard-Of-Care Treatment in Subjects With Multiple Myeloma, Glioblastoma Multiforme, and GI Malignancies | Cancer of Pancreas<br>Cancer of Liver<br>Cancer of Rectum<br>Cancer of Colon<br>Gall Bladder Cancer<br>Multiple Myeloma<br>Glioblastoma Multiforme | Drug: Cannabidiol<br>Drug: Bortezomib<br>Drug: Leucovorin<br>Drug: 5-FU<br>Drug: Oxaliplatin<br>Drug: Bevacizumab<br>Drug: Irinotecan<br>Drug: Gemcitabine<br>Drug: Temozolomide | Unknown | NCT03607643 |
| Safety | | | | |
| Assessment of Safety, Tolerability and Pharmacokinetics of Single Doses of Oral Dexanabinol in Healthy Subjects | Safety<br>Tolerability<br>Pharmacokinetics<br>Cancer | Drug: Dexanabinol Dose Level 1<br>Drug: Dexanabinol Dose Level 2<br>Drug: Dexanabinol Dose Level 3<br>Drug: Dexanabinol Dose Level 4<br>Drug: Dexanabinol Dose Level 5<br>Drug: Placebo | Completed (no publications) | NCT02054754 |
| An Observational Post-Marketing Safety Registry Of Patients Who Have Been Prescribed Sativex | Multiple Sclerosis<br>Diabetes<br>Cancer<br>Neuropathic Pain | Drug: Sativex | Completed (no results) | NCT02073474 |
